# A Route to Chaotic Behavior of Single Neuron Exposed to External Electromagnetic Radiation

**DOI:** 10.3389/fncom.2017.00094

**Published:** 2017-10-17

**Authors:** Peihua Feng, Ying Wu, Jiazhong Zhang

**Affiliations:** ^1^Department of Mechanics, School of Aerospace, Xi'an Jiaotong University, Xi'an, China; ^2^State Key Laboratory for Strength and Vibration of Mechanical Structures, Shaanxi Engineering Laboratory for Vibration Control of Aerospace Structures, School of Aerospace, Xi'an Jiaotong University, Xi'an, China; ^3^Key Laboratory for NeuroInformation of Ministry of Education, University of Electronic Science and Technology of China, Chengdu, China; ^4^Department of Fluid Machinery and Engineering, School of Power and Energy Engineering, Xi'an Jiaotong University, Xi'an, China

**Keywords:** neuron, chaos, electromagnetic radiation, transition of mode

## Abstract

Non-linear behaviors of a single neuron described by Fitzhugh-Nagumo (FHN) neuron model, with external electromagnetic radiation considered, is investigated. It is discovered that with external electromagnetic radiation in form of a cosine function, the mode selection of membrane potential occurs among periodic, quasi-periodic, and chaotic motions as increasing the frequency of external transmembrane current, which is selected as a sinusoidal function. When the frequency is small or large enough, periodic, and quasi-periodic motions are captured alternatively. Otherwise, when frequency is in interval 0.778 < ω < 2.208, chaotic motion characterizes the main behavior type. The mechanism of mode transition from quasi-periodic to chaotic motion is also observed when varying the amplitude of external electromagnetic radiation. The frequency apparently plays a more important role in determining the system behavior.

## 1. Introduction

Dynamic behaviors of single neuron or neural network are essential to understanding complex behaviors in brain or even serious diseases in nervous system. On account of complexity of nervous system, hundreds of equations should be set up to describe neuronal behavior precisely. However, main properties like electrical activities in neurons catch the attention of most researchers, and based on which, several models are established to physically study mode selection of neurons. For example, four-variable Hodgkin-Huxley (HH) equations are usually used to investigate the main properties of neurons via membrane potential (Hodgkin and Huxley, 1990). For further simplification, a three-variable neuron model, called as Hindmarsh-Rose (HR) equation, is reduced from the four-variable HH model (Hindmarsh and Rose, 1982). Besides ordinary differential equations, discrete dynamical systems or called maps, can also be considered as valid phenomenological neuron models to govern evolution of the transmembrane voltage and the dynamics of ionic conductances (Ibarz et al., 2011).

Among all these equations, Fitzhugh-Nagumo (FHN) neuron model shows its validity on oscillatory dynamic behavior in a neuron (Fitzhugh, 1961; Nagumo et al., 1962). The model presents properties of Van der Pol oscillator which can also be analyzed by fast- slow system (Krupa et al., 1997). Many methods are applied for constructing the exact solutions of the FHN equation (Li and Guo, 2006; Dehghan et al., 2010) to study transmission of nerve impulses. FHN equation is usually considered as a non-linear ordinary differential wave equation, and its soliton solutions are detected in different ways (Abbasbandy, 2008; Triki and Wazwaz, 2013). As the most important and complex system in nature, the brain contains a tremendous number of neurons and gliocytes. Collective behaviors of a large set of neurons and dynamics properties of neuron network are of much more importance. Patterns like spiral waves and targeted waves and their breakdown show the complexity of brain and related to horrible disease in neuron system. Sported, stripe, and hexagon patterns are also discovered in a modified FHN model which are very similar to the situation in reaction diffusion system.

Among all the factors affecting dynamic behaviors and pattern formation in neuron model, noise, and magnetic flow etc. are the most important and studied by many researchers. Different dynamical regimes are observed induced by external noise (Garcaojalvo and Schimanskygeier, 1999). Wu measures the pattern transition from subexcitable to excitable media (Ying et al., 2013). Colored noise can enhance the stochastic resonance in FHN neuronal model (Nozaki and Yamamoto, 1998). Noise could also be suppressed by a strong periodic signal (Pankratova et al., 2005). Besides, magnetic flux shows its high affection on collective electrical activities and signals propagation among neurons (Lv et al., 2016). Specifically, mode transition is detected and mismatch of frequency between electromagnetic radiation and the system is found (Ma et al., 2017). Magnetic flux on membrane potential is realized by a mimristor coupling, which leads to non-linear quasi-periodic spatial-temporal patterns (Mvogo et al., 2017).

As a matter of fact, a number of researchers are working on the effects on many facets of biological phenomena of electromagnetic radiation (Ma and Tang, 2017), such as central nervous system (Hossmann and Hermann, 2003), newborn rat cerebellar granule neurons (Lisi et al., 2005), oxidative damage to mitochondrial DNA in primary cultured neurons exposed to 1,800 MHz radio frequency radiation (Xu et al., 2010), cultured hippocampal neurons of rats exposed to microwave radiation (Xu et al., 2006). As to the neuron network, electromagnetic radiation can also play an important role in regulating collective behaviors among a large number of neurons. Usually, chemical and electric synapse is considered as the main type of connection between neurons. But in Ma's view, field coupling also lights the shadow of understanding synchronization problems in neuronal network (Ma and Tang, 2017). Dynamical features of neuron model with electromagnetic radiation considered should be paid more attention.

In spite of the fact that external magnetic flux is introduced into neuron network and it triggers complex patterns, the affection on a single neuron is still worth exploring deeply. Ma (Ma et al., 2017) develops FHN model and chooses a sinusoidal function as the external magnetic flux. There also exists a sinusoidal function as a transmembrane current mapped from external forcing in original model. The fact means the neuron system is driven by a pair of plane waves which leads to abundant non-linear behaviors in system. In order to compare with the results of Ma, we choose the same improved model, even with identical parameter values in reference paper (Ma et al., 2017). Model and numerical methods are explained in section 2. We provide and analysis the main numerical results when varying frequency of external transmembrane current ω in section 3, and when varying the amplitude of external electromagnetic radiation A in section 4, respectively. The conclusions are drawn in section 5.

## 2. Model description

The improved FHN model, with magnetic flux considered, is displayed as Equation (1).

(1) $$\left\{ \begin{array}{l} \frac{\text{d}u}{\text{d}t}=-k(u-a)(u-1.0)-uv+I_{st}+k_{0}\rho (\varphi )u\\ \frac{\text{d}v}{\text{d}t}=(\varepsilon +\frac{\mu_{1}v}{u+\mu_{2}})[-v-ku(u-a-1.0)]\\ \frac{\text{d}\varphi }{\text{d}t}=k_{1}u-k_{2}\varphi +\varphi_{ext}. \end{array}\right.$$

The additive magnetic flux is induced by changing the distribution of ionic concentration of lectrolytes. The evolution of magnetic flux across the membrane is described by the third equation in Equation (1), which has great effects on membrane potential u in first equation in Equation (1) by a induced current denoted by the last term *k*_0_ρ(φ)*u*. The memory conductance ρ(φ) of a memristor controlled by magnetic flux, which is often described by

(2) $$\rho (\varphi )=\alpha +3\beta \varphi^{2}$$

The second *v* is slow variable for current and magnetic flux across the membrane. The physical meaning of other parameters are present in the reference (Ma et al., 2017). It is important that both the external electromagnetic radiation φ_*ext*_ and the transmembrane current *I*_*st*_ are chosen as trigonometric functions like,

(3) $$\varphi_{ext}=A\text{ cos }2\pi ft$$

(4) $$I_{st}=I_{0}\text{ sin }\omega t$$

respectively. The system is driven by these two plane waves. As to simulation, we use fourth order Runge-Kutta algorithm and the time step is *h* = 0.01. We choose the same initial values (*u, v*, φ) = (0.2, 0.1, 0.8). Values of other parameters are listed here, *a* = 0.15, μ_1_ = 0.2, μ_2_ = 0.3, ε = 0.002, α = 0.1, β = 0.2, *I*_0_ = 0.6, *k*_0_ = −1, *k*_1_ = 0.2, *k*_2_ = 1.0.

## 3. Main numerical results as varying angular frequency ω

### 3.1. Main types of motions of membrane electrical behaviors

Varying transmembrane current *I*_0_ could lead to transformation of mode of electrical activities. However, in this section we focus on ω as our control parameter in system. Ma (Ma et al., 2017) already discovered different types of dynamic behavior like periodic and chaotic-like motions and even bursting phenomenon in the sampled time series for membrane potentials. As matter of fact, periodic, quasi-periodic, and chaotic motions are all discovered in this situation. The parameters related to the external electromagnetic radiation are selected by *A* = 0.1 and *f* = 0.01 in this section.

**Figure 1:**
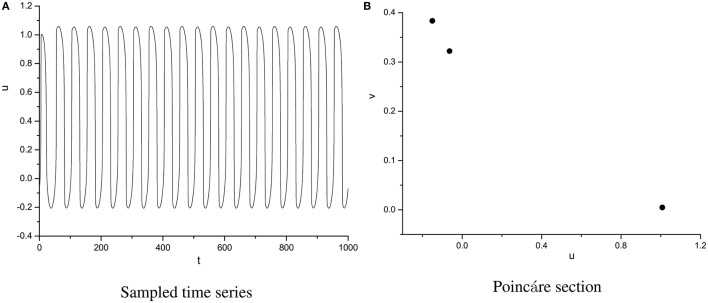
Sampled time series **(A)** and its Poincáre section **(B)** as ω = 0.1256.

**Figure 2:**
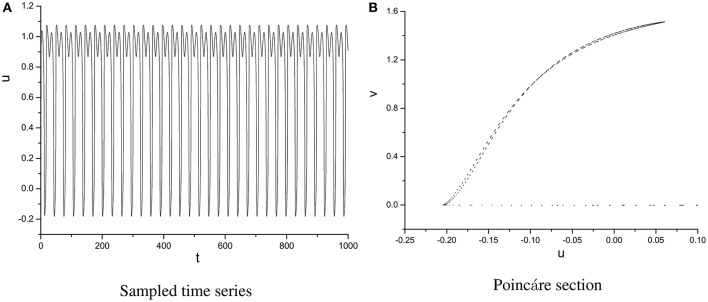
Sampled time series **(A)** and its Poincáre section **(B)** as ω = 0.4.

**Figure 3:**
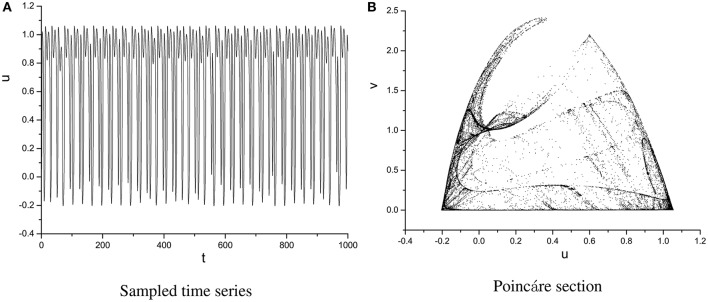
Sampled time series **(A)** and its Poincáre section **(B)** as ω = 0.6.

**Figure 4:**
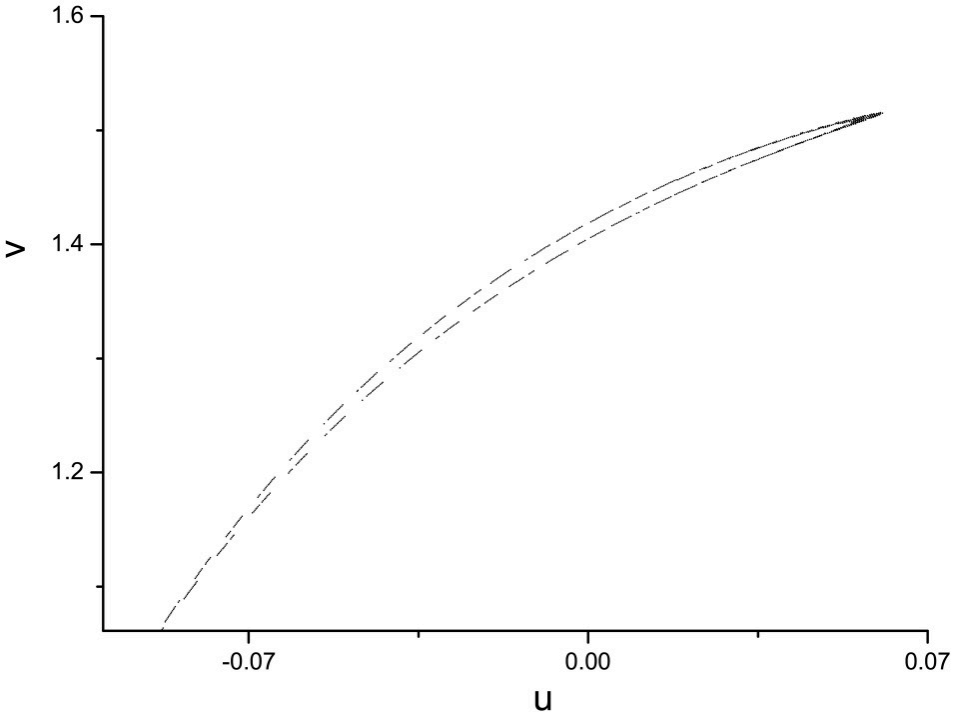
Enlarged view of Figure 2B, A close circle in Poincáre section as ω = 0.4.

In order to specify the different types of behavior, Poincáre section φ = 0 is used. Poincáre section can help us not only estimate the motion types but also decide the value of number N of Period-N when the motion is periodic (Song et al., 2015a,b, 2016). Sampled time series for membrane potential and their Poincáre sections are plotted in Figures 1–3 as ω = 0.1256, ω = 0.4, and ω = 0.6, respectively. According to Poincáre section, three isolated points are displayed in Figure 1B, which means the dynamic behavior when ω = 0.1256 is periodic motion. Closed orbit in Poincáre section is shown in Figure 2B, corresponding to the quasi-periodic motions as ω = 0.4, which is zoomed in Figure 4 more clearly. When ω reaches up to ω = 0.6, the closed orbit is destroyed and certain structure appears in Poincáre section, shown in Figure 3B.

**Figure 5:**
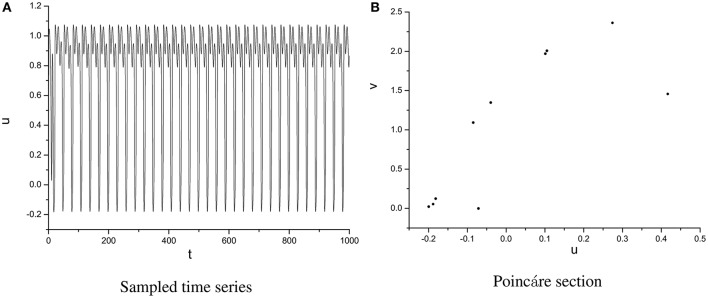
Sampled time series **(A)** and its Poincáre section **(B)** as ω = 0.618.

**Figure 6:**
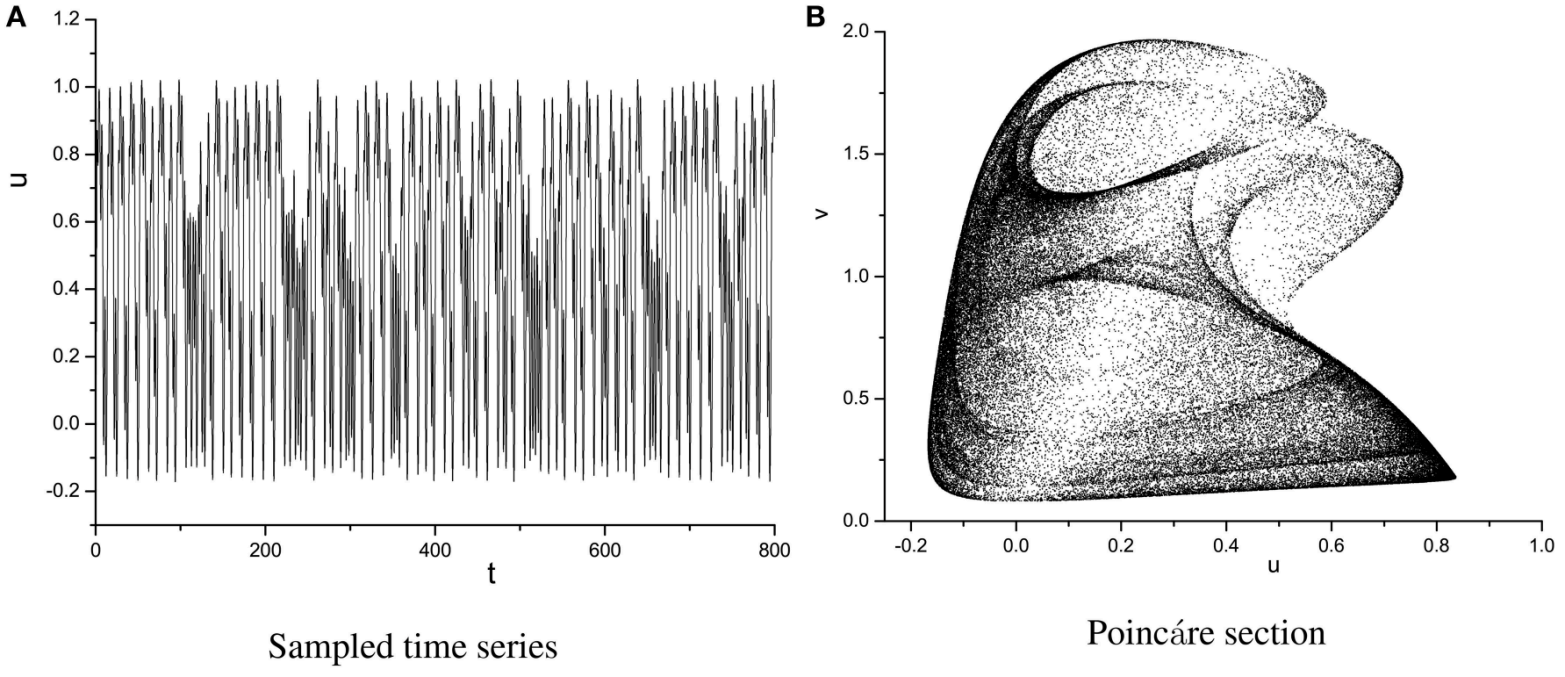
Sampled time series **(A)** and its Poincáre section **(B)** as ω = 2.0.

**Figure 7:**
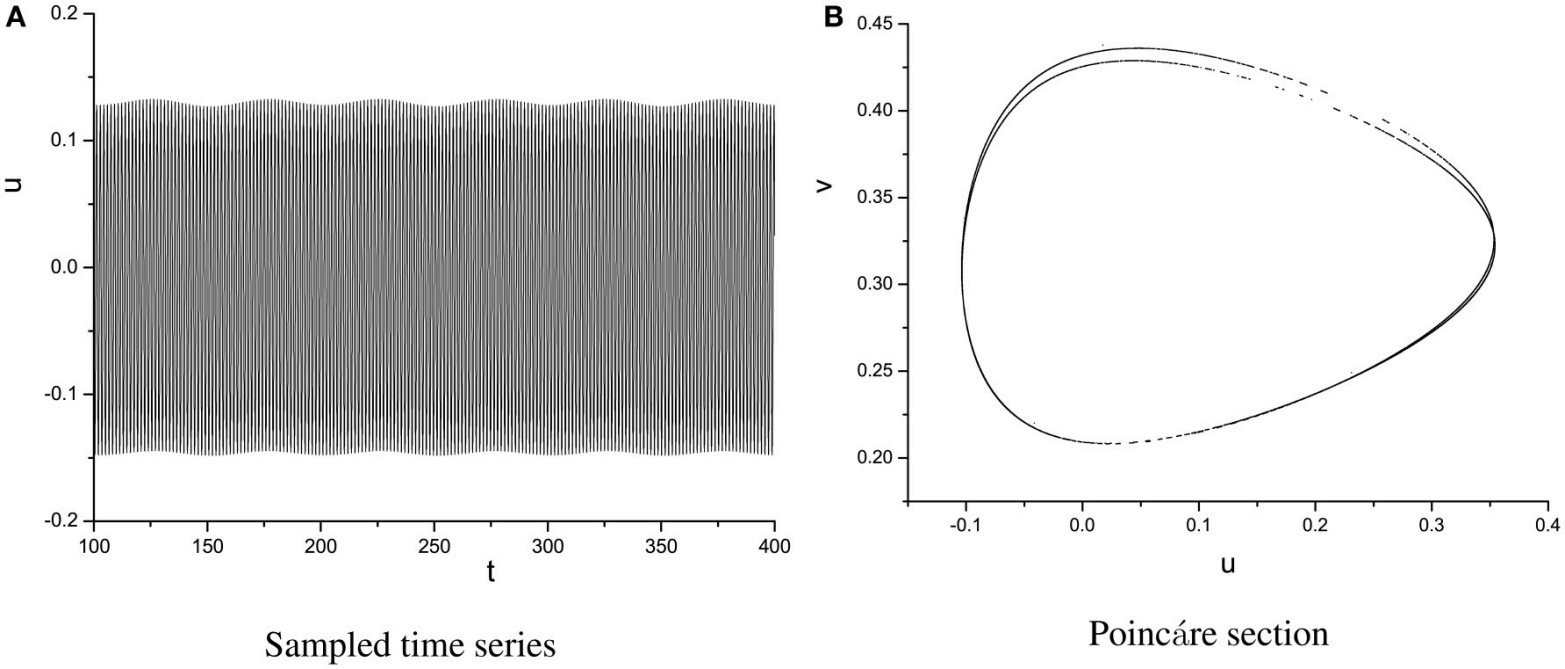
Sampled time series **(A)** and its Poincáre section **(B)** as ω = 4.0.

Furthermore, if we keep going to increase ω, quasi-periodic, periodic, and chaotic motions can also be captured, which are plotted in Figures 5–7. Discrete points, strange structures, and closed circles are displayed in Poincáre section, respectively.

### 3.2. Modes selection to angular frequency ω

It seems that motion of system changes irregularly when we increase angular frequency ω. In order to investigate mode selection, mode transition process is studied carefully. Usually, inter-spike interval (ISI) (Duan et al., 2008; Wang et al., 2014) is an effective tool to analysis the bifurcation of system, and it is also shown in reference (Ma et al., 2017). Also, it is easy to use ISI diagram to describe the multi-mode of electrical activities. Hierarchy in ISI diagram means the different discharge process. However, it is difficult to estimate the type of motion in ISI diagram. We propose another diagram to distinguish different types of motion. We plot the second variable *v* appearing its corresponding Poincáre section under every parameter value. A few discrete points can be observed under certain parameter value if the motion is periodic. At least it is effective to distinguish the periodic motion from quasi-periodic and chaotic motions. We use nomenclature of semi-Poincáre diagram to call this method to describe transition of motion types.

**Figure 8:**
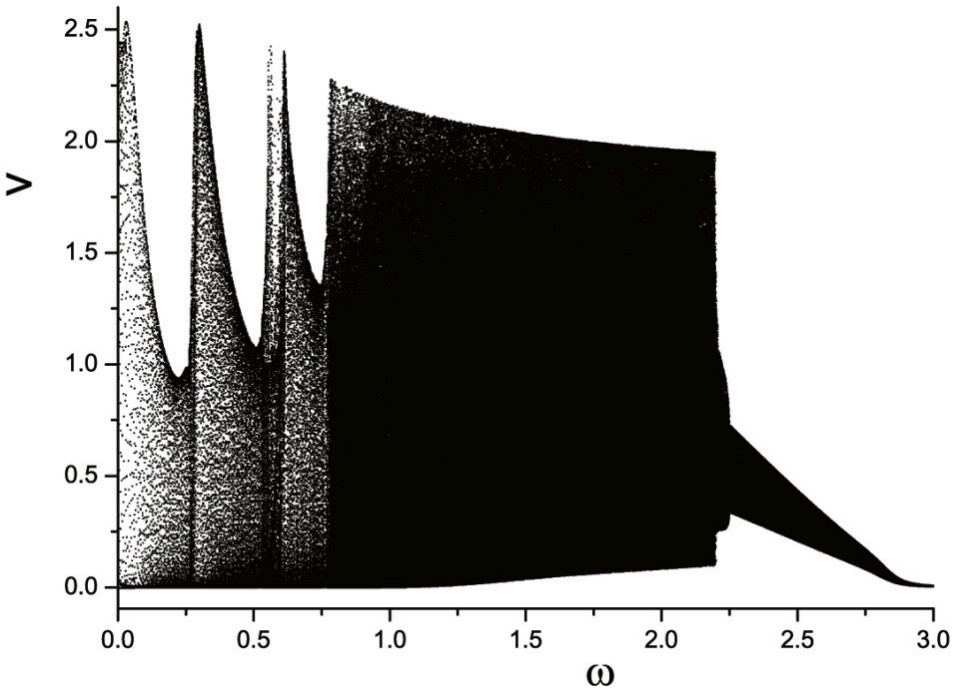
Semi-Poincáre diagram when varying ω during (0, 3.0).

**Figure 9:**
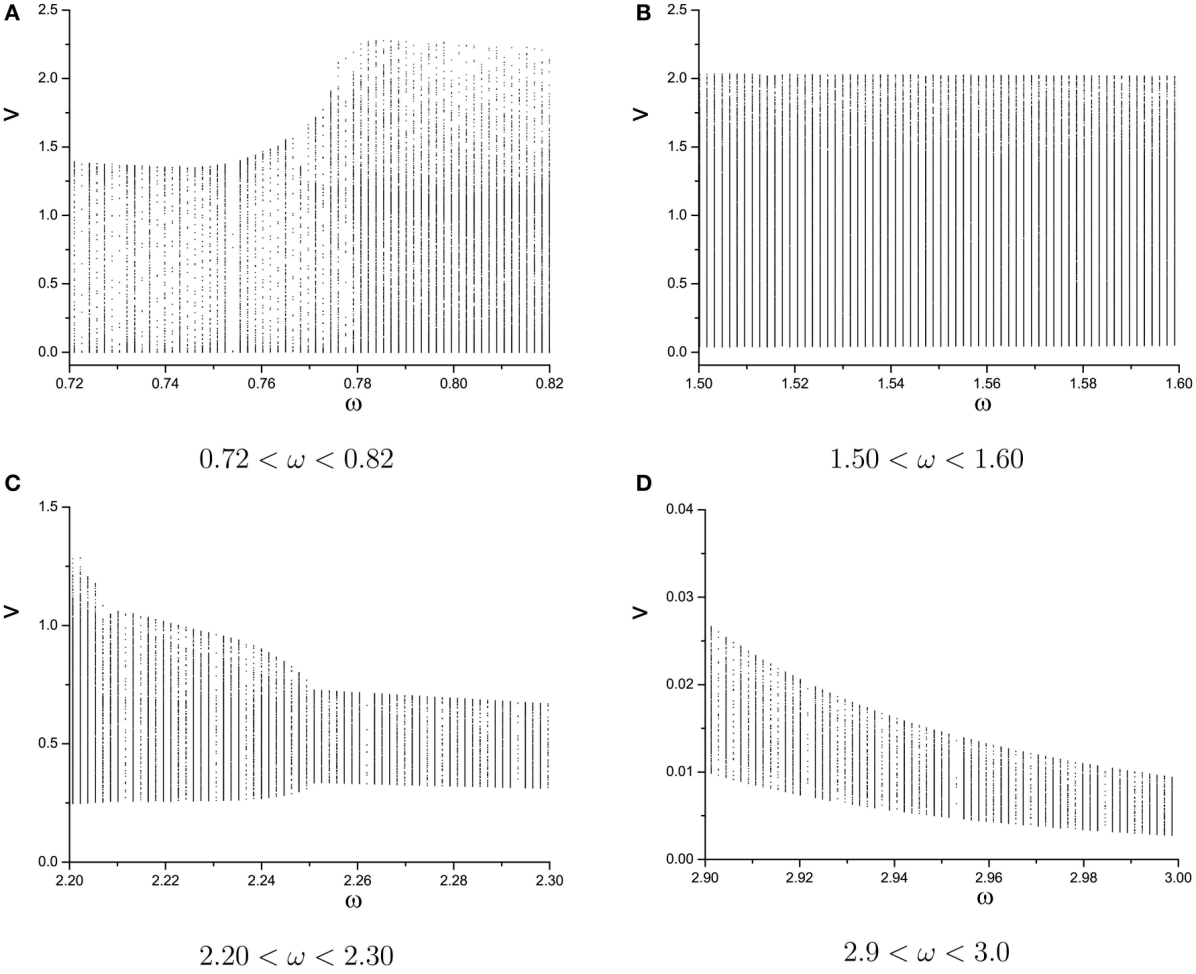
Enlarged views of semi-Poincáre diagram in different ω intervals. **(A)** 0.72 < ω < 0.82, **(B)** 1.50 < ω < 1.60, **(C)** 2.20 < ω < 2.30, and **(D)** 2.9 < ω < 3.0.

**Figure 10:**
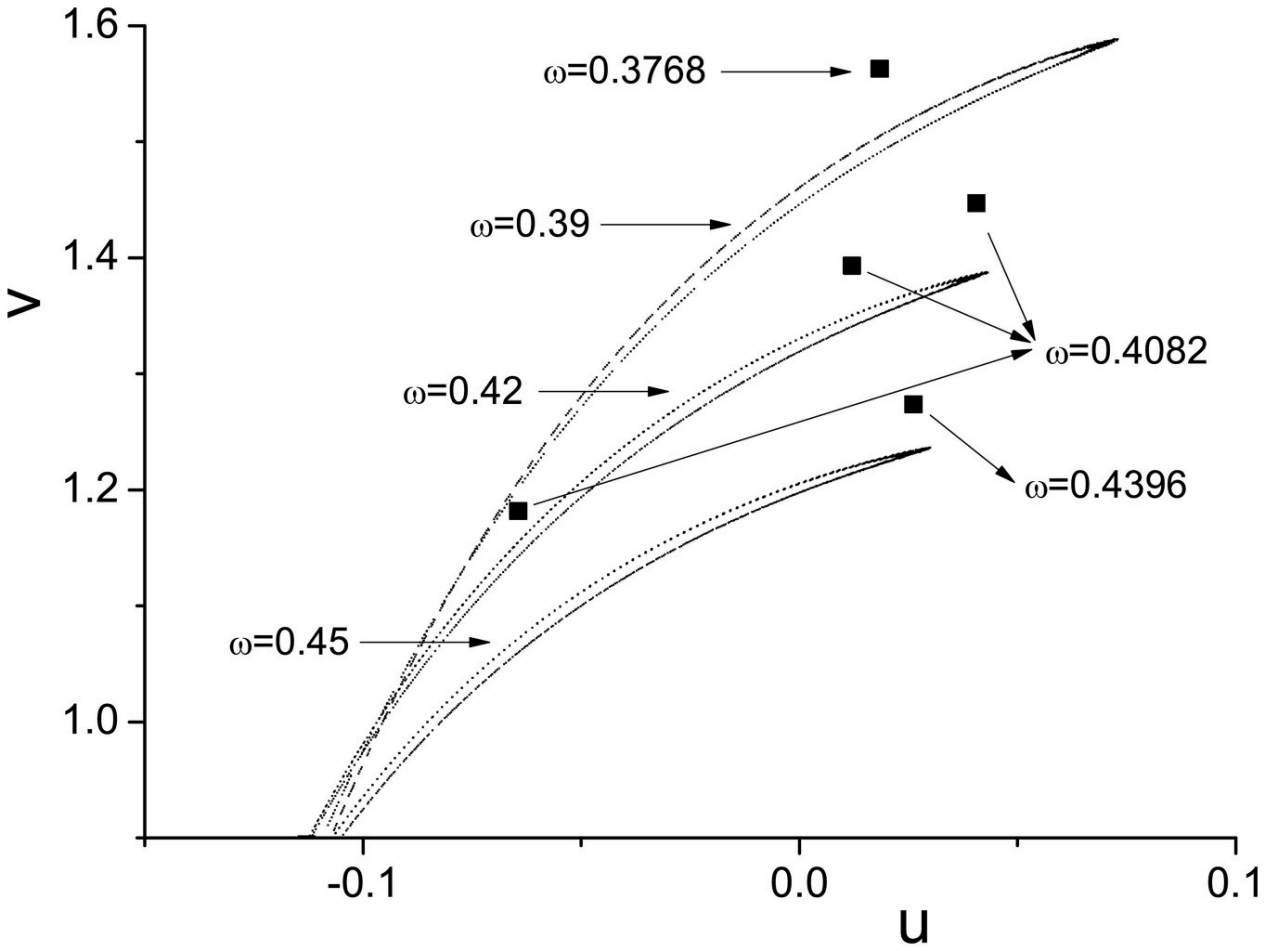
Poincáre sections at different ω. Transition of motion type when changing ω.

We plot the semi-Poincáre diagram with 0 < ω < 3.0, which is shown in Figure 8. Enlarged view of Figure 8 in different ω intervals is shown in Figure 9. Discrete points and continuous lines distribute alternatively. Even slight change of ω can lead to great change of motion type. Poincáre section as ω is around 0.4 is shown in Figure 10. Discrete points and closed circles appear in Poincáre section alternatively, which means that type of motion switches back and forth between periodic and quasi-periodic motion. But according to Figure 9A the scenario of switching between the two motion types is interrupted by chaotic behaviors. The motion type of system becomes chaotic when ω is >0.778. and it lasts until ω = 2.208. One of ω interval of chaos characterized by continues lines is shown in Figure 9B. In other words, in the interval 0.778 < ω < 2.208, periodic and quasi-periodic motions disappear and only chaotic motion is captured. However,when ω > 2.208 the system goes back to the situation of switching back and forth between periodic and quasi-periodic motions, shown in Figures 9C,D. But the scenario is still very different from that in interval ω < 0.778. specifically, compared to situation of ω < 0.778, quasi-periodic motion dominates the interval ω > 2.208 and the chance of occurrence of periodic motions is small. It is highly likely that quasi-periodic motion is interrupted by periodic motion occasionally.

**Figure 11:**
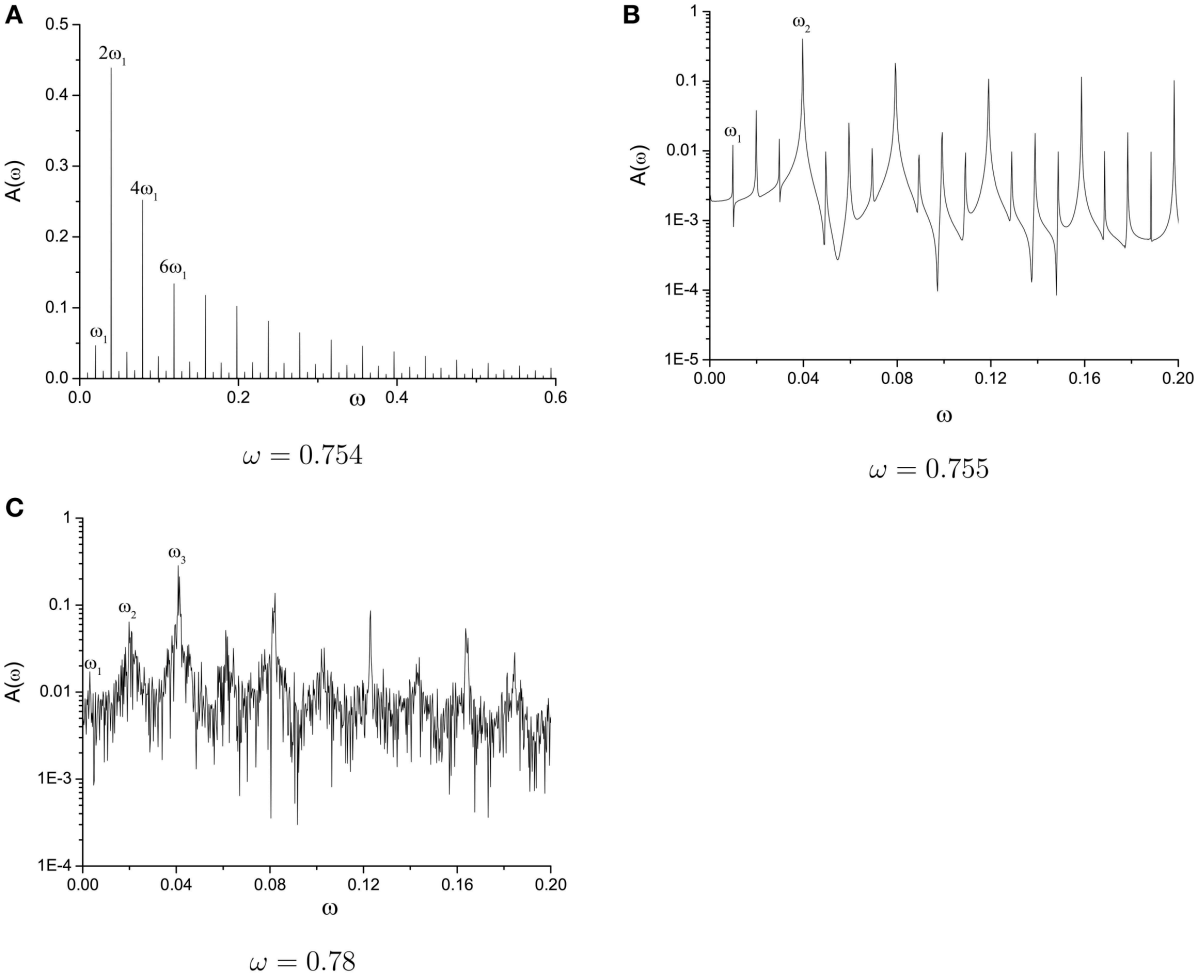
Frequency spectrum of second variable for the three different motion types. **(A)** ω = 0.754, **(B)** ω = 0.755, and **(C)** ω = 0.78.

Hopf bifurcation plays the key role in motion type selection. Irreducible frequency introduced into system by Hopf bifurcation leads the system from periodic motion to quasi-periodic motion or from quasi-periodic to chaos. In order to specify the statement, we also provide the frequency spectrum of second variable for the three different motion type when the parameter ω is closed to each other. We select ω = 0.754, ω = 0.755, and ω = 0.78 for periodic, quasi-periodic, and chaotic motion types. One, two, and three irreducible frequencies are discovered in frequency spectrum diagram (see Figure 11). New irreducible frequency generated via Hopf bifurcation brings the discrete discrete spectrum to continuous spectrum of chaotic motion. The route to chaos of this kind by a few Hopf bifurcations is called Ruelle-Takens route to chaos (Ruelle and Takens, 1971). A series of supercritical and subcritical Hopf bifurcations lead to the transition of different motion types alternatively.

## 4. Main results as varying the amplitude of external electromagnetic radiation A

External electromagnetic radiation is chosen as a cosine function φ_*ext*_ = *A* cos 2π*ft*. The amplitude A is also an important control parameter, which decides the state of system when other parameters are fixed. We still use semi-Poincáre diagram to describe the scenario as varying A during 0 < *A* < 1.0. The situation of *A* > 1.0 will not shown in this paper because the the system stay in equilibrium state, which means all the variables remain constants.

**Figure 12:**
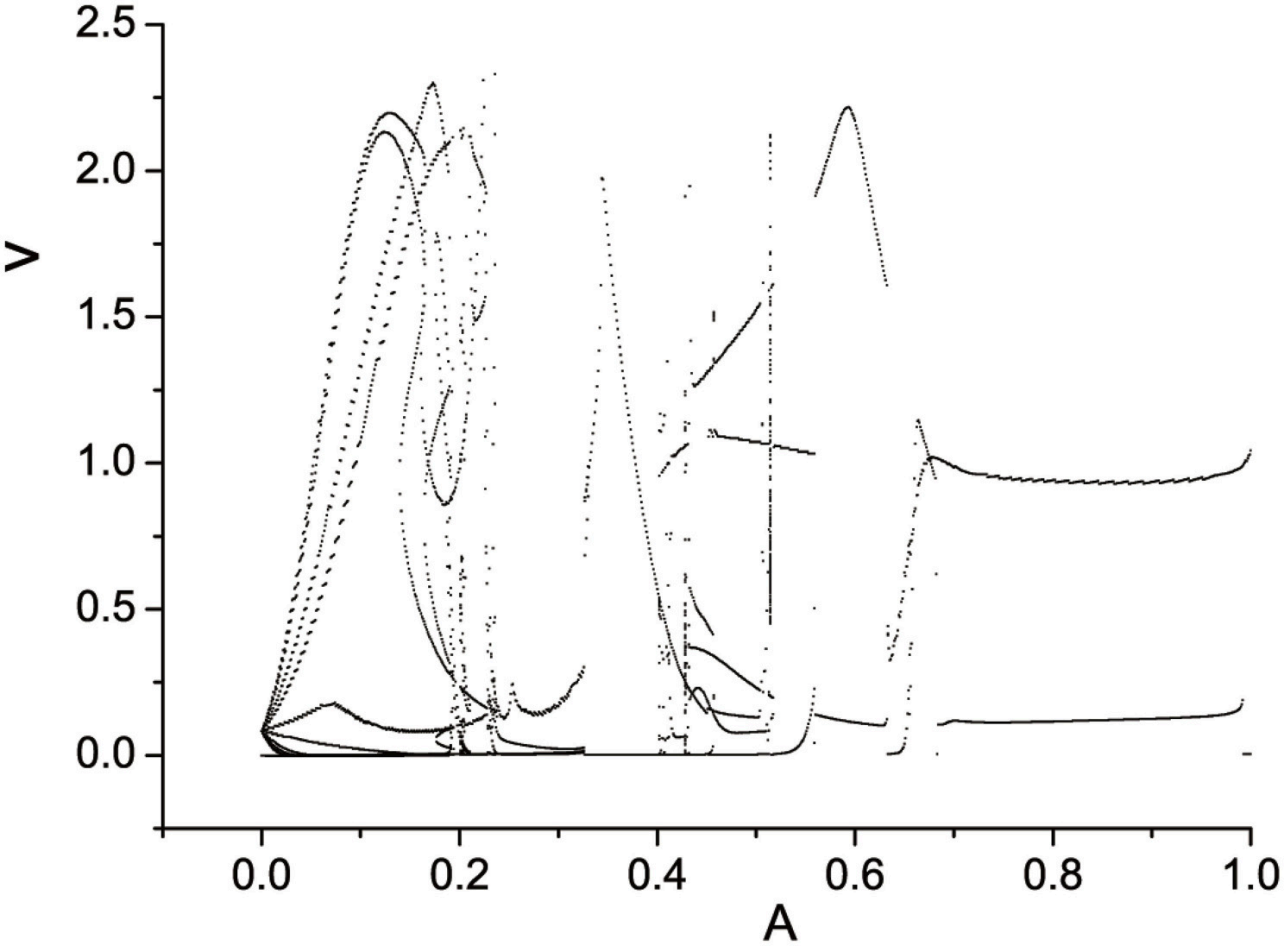
Semi-Poincáre diagram as A through (0, 1.0) when ω = 0.618.

**Figure 13:**
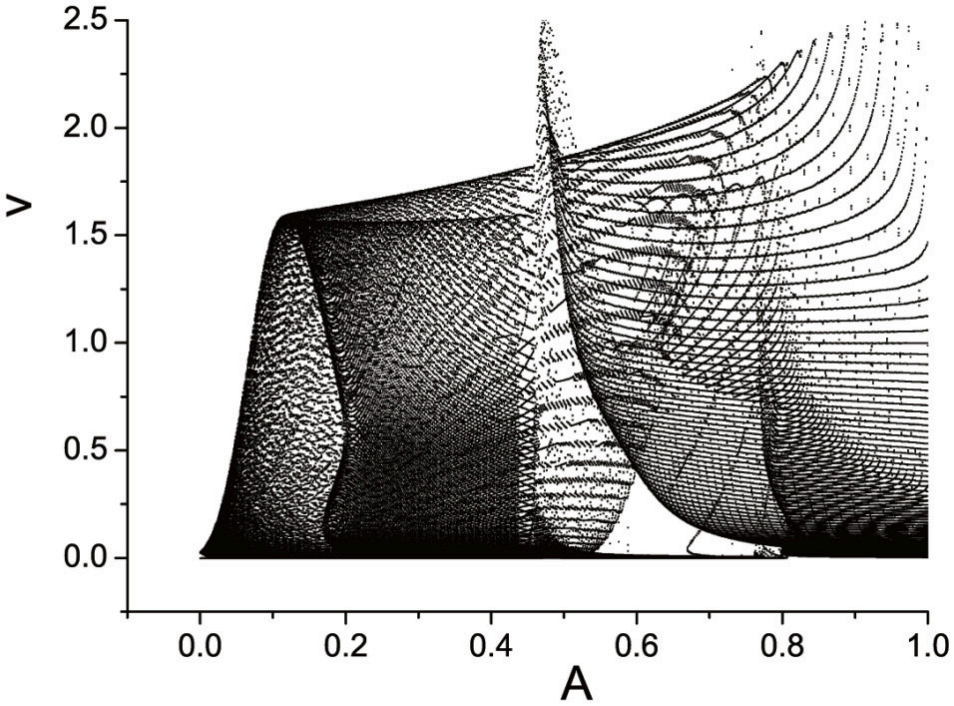
Semi-Poincáre diagram as A through (0, 1.0) when ω = 0.4.

**Figure 14:**
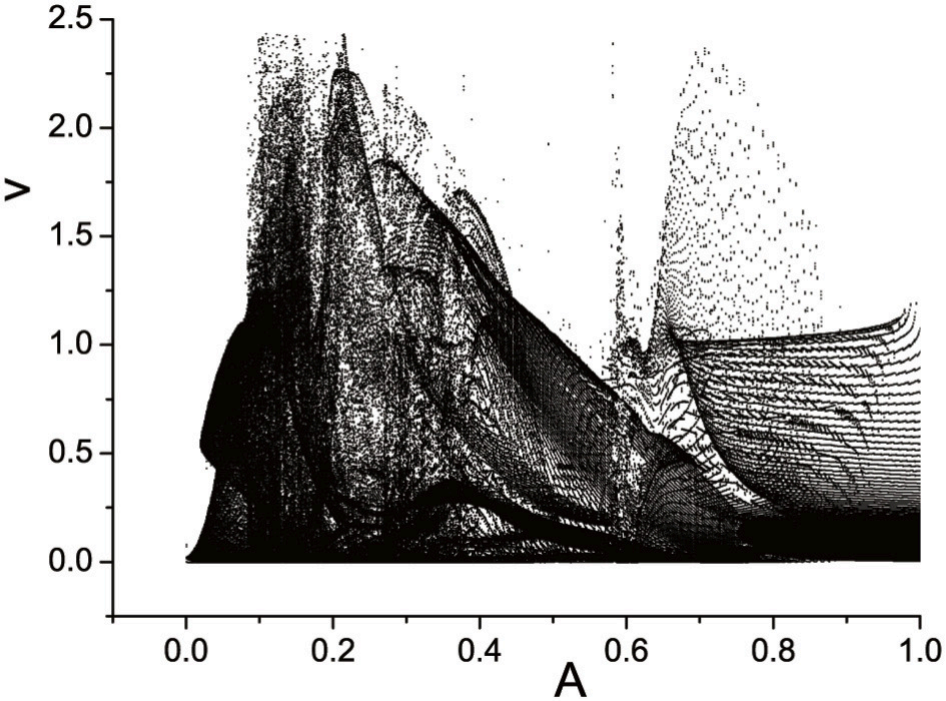
Semi-Poincáre diagram as A through (0, 1.0) when ω = 0.6.

Three semi-Poincáre diagrams are plotted as ω = 0.618, ω = 0.4, and ω = 0.6, corresponding to periodic, quasi-periodic, and chaotic motion when A is fixed in 0.1, shown in Figures 12–**14**, respectively.

In large span of 0 < *A* < 1.0, periodic motion is discovered, but is is interrupted by quasi-periodic motion several times, when ω = 0.618 (Figure 12). When A is fixed at 0.4, it seems that quasi-periodic motion is more easy to be captured during the interval 0 < *A* < 1.0 (Figure 13). Similarly, the chance we find chaotic behavior is much more than periodic and quasi-periodic motions when ω = 0.6, which can be reflected by Figure 14.

**Figure 15:**
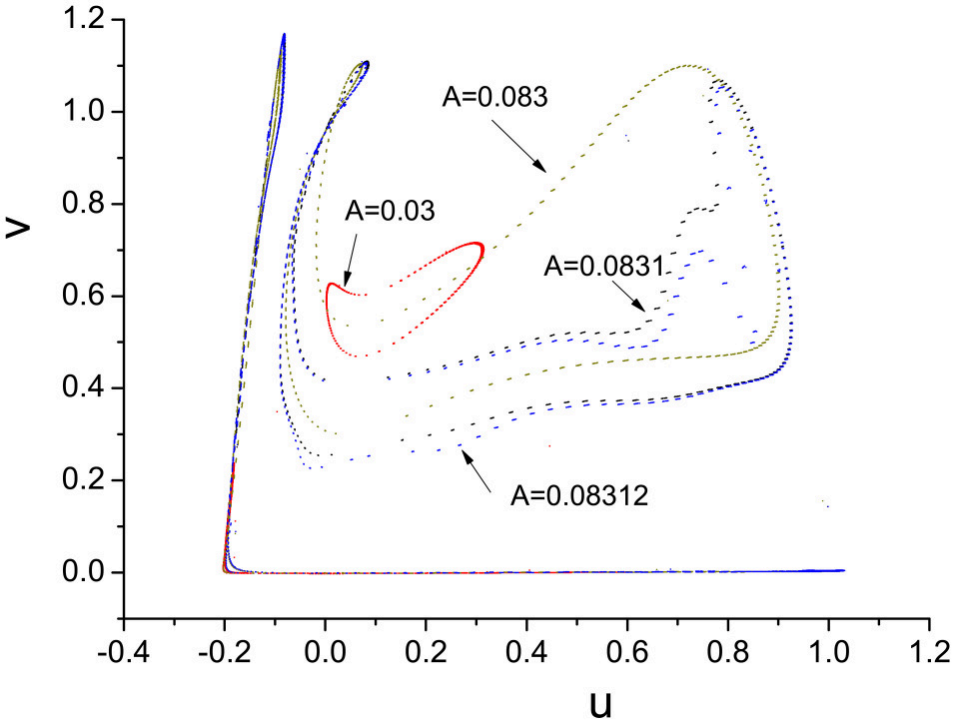
Process of growth of the orbit in Poincáre section when ω = 0.6.

It is worth noting that Ruelle-Takens route to chaos also exists when varying the parameter A. Take the scenario shown in Figure 14 for example, there are only two closed orbits in Poincáre section when *A* < 0.0019, however, three closed orbits appear when A is >0.0019. The new orbit is growing lager when we increase A. The process of growth of the new orbit is stopped by a Hopf bifurcation and system enters the chaotic state when A is >0.0832. The growth process of orbit in Poincáre section is shown in Figure 15.

## 5. Conclusions and discussion

In this paper, we study dynamical behavior of a single neuron system driven by two plane waves. One of them is provided by external current denoted by *I*_*st*_, and another comes from the external electromagnetic radiation φ_*ext*_.

With frequency of electromagnetic radiation φ_*ext*_ fixed, system state is chosen from periodic, quasi-periodic, and chaotic motion. Specifically, system behavior switches back and forth between periodic and quasi-periodic when 0 < ω < 0.778. In the interval of 0.778 < ω < 2.208, only chaotic motion is fond. But when ω is beyond 2.208, the system alternates again between periodic and quasi-periodic motions and the chance of finding quasi-periodic motion is much more than 0 < ω < 0.778.

We also study the non-linear behavior of system varying amplitude of electromagnetic radiation A at three ω values. Very different scenarios are displayed. Periodic, quasi-periodic, or chaotic motion type characterize the scenario at different ω value, respectively.

The route to chaos, induced by Hopf bifurcation, is discovered varying both frequency ω of electromagnetic radiation φ_*ext*_ and amplitude of electromagnetic radiation A. However, it seems ω plays a much more important role in controlling the system state. In summary, system behavior is very sensitive to frequency of external force, which might be related to the ratio of frequencies of two external plane waves.

## Author contributions

All authors listed have made a substantial, direct and intellectual contribution to the work, and approved it for publication.

### Conflict of interest statement

The authors declare that the research was conducted in the absence of any commercial or financial relationships that could be construed as a potential conflict of interest.
